# Neuroinflammation Markers in Depressive Female Adolescents with Suicidal Attempts

**DOI:** 10.1192/j.eurpsy.2022.648

**Published:** 2022-09-01

**Authors:** A. Iznak, E. Iznak, E. Damyanovich, I. Oleichik, S. Zozulya

**Affiliations:** 1 Mental Health Research Centre, Laboratory Of Neurophysiology, Moscow, Russian Federation; 2 Mental Health Research Centre, Department Of Endogenous Mental Disorders And Affective Conditions, Moscow, Russian Federation; 3 Mental Health Research Centre, Laboratory Of Neuroimmunology, Moscow, Russian Federation

**Keywords:** suicidal attempts, neuroinflammation, Adolescents, quantitative EEG

## Abstract

**Introduction:**

Suicide is the second leading cause of death (8.5% of all deaths) in adolescents. The search for neurobiological markers of suicidal behavior seems to be highly actual. Such markers may include quantitative EEG parameters and signs of neuroinflammation that plays an important role in the pathogenesis of various mental disorders.

**Objectives:**

The aim of the study was to reveal the relationships between pre-treatment clinical, EEG, and neuroimmunological parameters in depressive adolescents with suicidal attempts in their history.

**Methods:**

35 female depressive patients (all right-handed, age 16–25, mean 18,7±2.9 years old) were enrolled in the study. Total HDRS-17 scores varied from 13 to 43 (mean 27,7±8.1). Multichannel resting EEG was recorded with spectral power (SP) measurements in narrow frequency sub-bands. Functional activities of leukocyte elastase (LE) and of its antagonist α1-proteinase inhibitor (α1-PI), as neuroinflammation markers, were measured in the blood plasma. Leukocyte/inhibitory index (LII=LE/α1-PI) was calculated. Spearman’s correlations between clinical, EEG, and neuroimmunological parameters were analyzed.

**Results:**

Sum of anxiety cluster of HDRS-17 scale (items 9, 10, 11) correlated positively (p<0.02) with LE and α1-PI values, as well as with theta1 (4-6 Hz) and theta2 (6-8 Hz) SP in EEG leads of the right hemisphere. In turn, α1-PI values correlated negatively and LII values correlated positively with alpha3 (11-13 Hz) SP in majority of EEG leads.

**Conclusions:**

The data obtained confirm the contribution of neuroinflammation to clinical conditions, especially to anxiety level, and to EEG pattern in depressive female adolescents with suicidal attempts. The study supported by RBRF grant No.20-013-00129a.

**Disclosure:**

No significant relationships.

